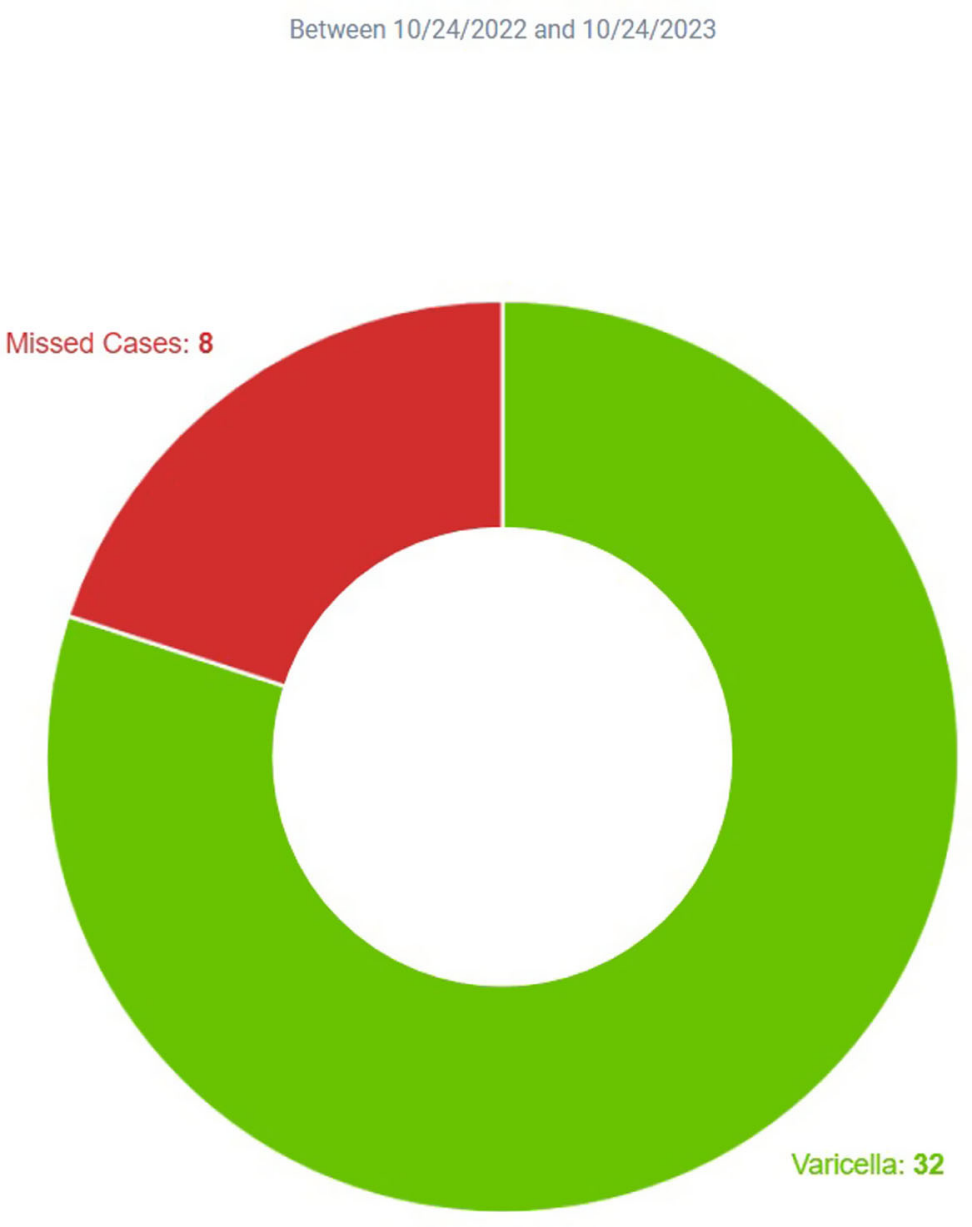# Utilizing Technology to Fill the Ambulatory Care Communicable Disease Practice Gap

**DOI:** 10.1017/ash.2024.305

**Published:** 2024-09-16

**Authors:** Amy Cook, Kyle Delp, Jennifer Laughman

**Affiliations:** WellSpan Health

## Abstract

**Background:** Infection Prevention (IP) practices in ambulatory care are often reactive and many communicable diseases in the community often do not fall onto IP’s radar until the patient becomes ill enough to seek inpatient services. The gap between ambulatory and inpatient care can lead to increased transmission and illness severity. Early identification has substantial impacts on timely implementation of IP mitigation strategies, appropriate handoff upon entry into other care settings, and timely reporting to public health organizations. Current IP processes underutilize electronic health record (EHR) capabilities by relying upon lab driven notifications. This project sought to redesign the IP’s workflows, advancing the health system beyond the acute care setting and into the ambulatory care setting by adding the power of diagnosis codes to close a practice gap. **Method:** Infection Prevention and Information Technology collaborated to build silent best practice advisories (BPAs) in the EHR that utilized diagnosis codes related to Varicella zoster (VZV) infection charted by ambulatory care providers. These BPAs function by triggering infection statuses that populate the patient chart and include instructions to front line staff on personal protective equipment (PPE) requirements. These BPAs also trigger real-time notifications to the IP team to determine the validity of the infection status and exposure work-up necessity. Chart reviews of diagnosis codes utilized in pre and post intervention timeframes were completed to understand the impact of the silent BPAs. Percentages of patients captured, and Healthcare Worker (HCW) exposure reviews reported were evaluated to demonstrate effectiveness. Plan, Do, Study, Act (PDSA) was utilized to respond to gaps, increase efficiency, and limit erroneous infection statuses. **Result:** The one-year pre-intervention period revealed 36 total diagnosis codes used for Varicella zoster (VZV) infection; 3% of these infection cases were captured and 4% of eligible cases were reported for HCW exposure review. The one-year post intervention period revealed 40 total diagnosis codes used for VZV infection; 80% of these infection cases were captured and 70% of eligible cases were reported for HCW exposure review. PDSA quality improvement cycles allowed for refinement of the BPA logic that further increased infection cases captured to 100% with 100% of eligible cases reported for HCW exposure review. **Conclusion:** Utilizing the EHR, the organization appreciated enhanced identification of patients in real-time with VZV infection that allowed for appropriate mitigation strategies to be implemented. This proactive workflow design helps minimize the risk of transmission between ambulatory and acute settings and facilitated HCW exposure reviews.

**Figure:**
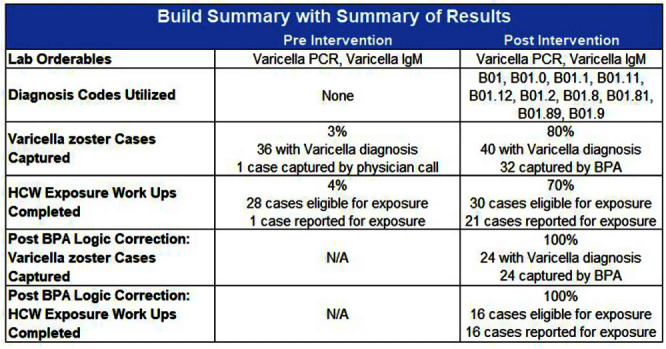

**Figure:**
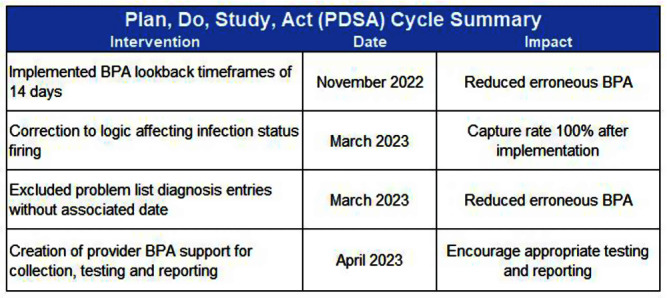

**Figure:**
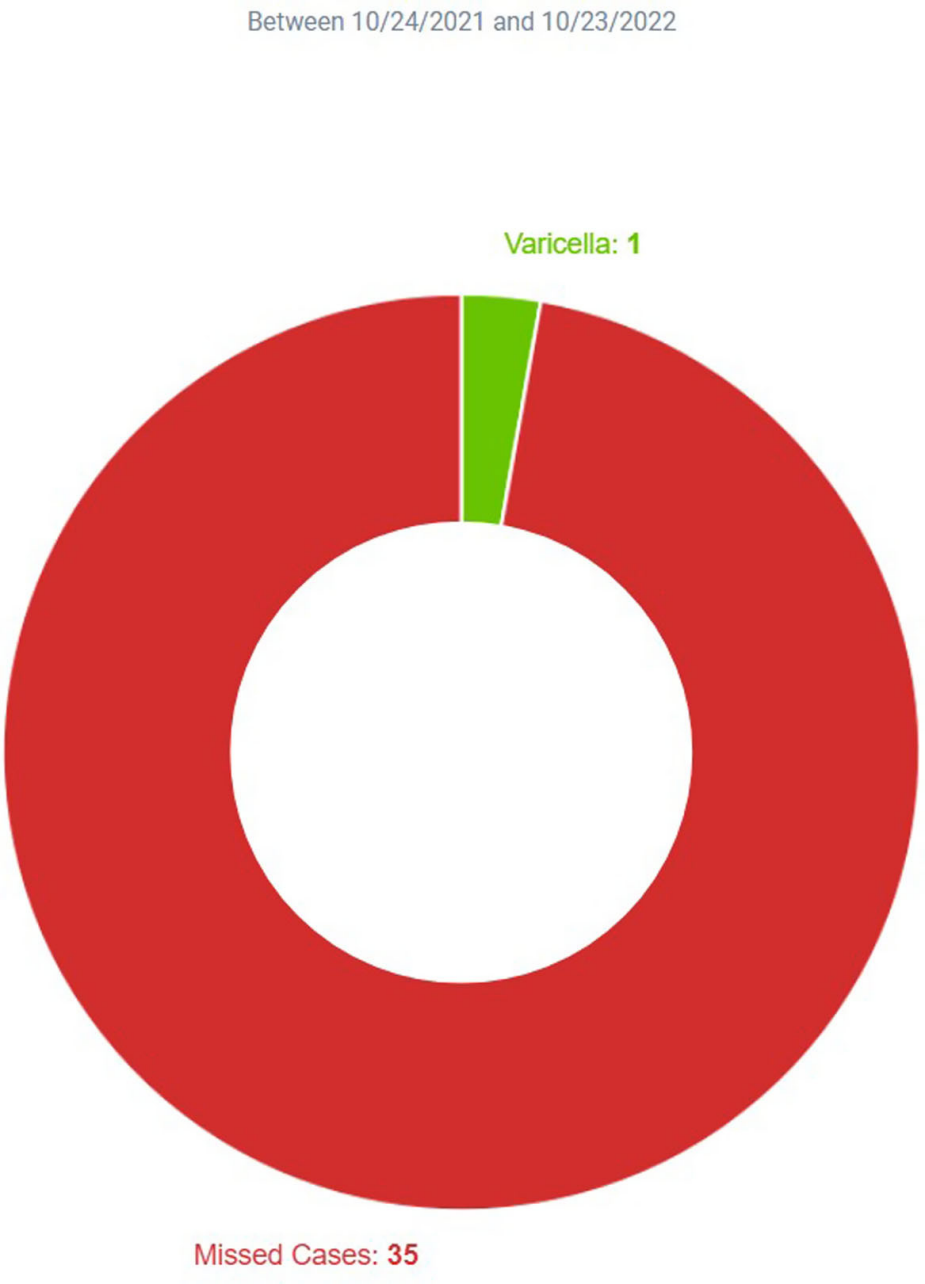

**Figure:**